# Investing in the Advanced Practice Nursing Workforce to Improve Health System Responses to Armed Conflict

**DOI:** 10.1111/inr.70074

**Published:** 2025-07-18

**Authors:** Joshua Porat‐Dahlerbruch, Jocelyn Boyd, Hilla Fighel

**Affiliations:** ^1^ Department of Acute and Tertiary Care University of Pittsburgh School of Nursing Pittsburgh Pennsylvania USA; ^2^ University of Pittsburgh School of Nursing Pittsburgh Pennsylvania USA; ^3^ Nursing Division Israel Ministry of Health Jerusalem Israel

**Keywords:** Advanced practice nursing, armed conflicts, delivery of healthcare, health workforce, nurse practitioner, public health infrastructure

## Abstract

**Aim:**

To pose an argument for health systems to improve responses to armed conflict by investing in developing the advanced practice nursing workforce.

**Background:**

Armed conflict catalyzes infectious disease, noncommunicable disease, and physical and psychological trauma. Health systems in countries at war face challenges in meeting the health service needs for affected populations while providing sufficient care for the rest of the public. Integrating advanced practice nurses into the workforce is one solution to address the demand for health services during war. Importantly, policies facilitating a quick, efficacious advanced practice nurse response during war must be in place before armed conflict arises.

**Sources of Evidence:**

A critical narrative review of peer‐reviewed articles was conducted. The review focused on the benefits of advanced practice nurses during crises and systemic policy setbacks preventing advanced practice nurse workforce development. A case study from the Israel–Hamas War is presented.

**Discussion:**

Holistic, patient‐centered care positions advanced practice nurses to care for the physical and psychological needs of the population arising from war. Evidence from other public health crises, such as the COVID‐19 and H1N1 pandemics, reinforces this notion. However, common policy setbacks, such as an ill‐defined scope of practice and a lack of interprofessional awareness of the role, prevent advanced practice nurses from providing care when conflict arises. Israel's policy efforts before and during the Israel–Hamas War exemplify advanced practice nursing workforce policy development that facilitated an effective response to crisis.

**Conclusion:**

Addressing expanded advanced practice nurse authorities before conflict starts will facilitate an improved health system response.

**Implications for Health Policy:**

Research examining the effectiveness of advanced practice nursing care quality during armed conflict will facilitate national‐level investment in advanced practice nursing workforce development.

## 1 Background

War is a catalyst for public health disasters. Armed conflict in the 21st century has been linked to increases in several health concerns (Garry and Checchi 2020). Beyond trauma‐induced injuries and mortality resulting from combat, there are additional long‐term health impacts. The incidence and prevalence of chronic noncommunicable diseases persist after wartime (e.g., cardiovascular and chronic respiratory disease) (D'Souza et al. 2022). Furthermore, there are secondary public health consequences resulting from the impact of war. As one example, infectious disease burden increases because of the displacement of residents to new locations and poor living conditions stemming from the economic impact of war (Marou et al. 2024). As a result of displacement and redistributing healthcare resources to the frontlines, sexual and reproductive health status declines from interruptions to routine antenatal and maternal care (Hedström and Herder 2023). Further, pediatric care is often not prioritized during wartime and can lead to an increase in under‐5‐year mortality, particularly in less wealthy nations (Jawad et al. 2023). Due to economic and trade disruptions during wartime, nutritional status tends to decline, which can result in downstream health effects (Acharya et al. 2020). Finally, long‐term mental health issues (e.g., posttraumatic stress disorder, anxiety, insomnia, and depression) arise in both military and public populations (Pavlova and Rogowska 2023). Effectively responding to the public health impact of armed conflict requires a system‐level response to manage the issues “upstream” (Jawad et al. 2020).

Health systems, at times of peace, already face challenges allocating scarce resources to deliver care efficaciously and equitably (Kruk et al. 2018). One example of a scarce resource is the healthcare workforce. During times of war, clinicians must deliver care to an increasing number of people, in new settings, and for newly pervasive health problems (Kamineni 2019). All the while, clinicians are tasked with maintaining standards of care quality (Kamineni 2019). Innovative workforce solutions, such as introducing advanced practice nurses (APNs), could better prepare nations for care during wartime.

The International Council of Nurses (2020, p. 6) defines an APN as “a generalist or specialized nurse who has acquired, through additional graduate education, the expert knowledge base, complex decision‐making skills, and clinical competencies shaped by the context in which they are credentialed to practice.” APNs deliver biopsychosocial, holistic patient and community‐level care (Rosa et al. 2020), which has improved care access and reduced morbidity during public health emergencies (Reed and Fitzpatrick 2024). APN roles “can be optimized to narrow disparities and ensure access to high‐quality health care globally” (Rosa et al. 2020, p. 554). However, it must be noted that not every population, particularly those displaced during wartime, has access to care from APNs and other providers (Porat‐Dahlerbruch et al. 2023a). As such, the burden falls on health systems to position and integrate APNs to maximally enhance their impact on populations most affected by armed conflict.

Several countries, either at war or on the brink of war, such as Israel and Taiwan, have introduced APN roles (Porat‐Dahlerbruch et al. 2023a). In Israel, APNs have helped meet provider demand throughout the Israel–Hamas War, which demonstrates a potential exemplar for other countries preparing their health system to provide care during crises. In contrast, Ukraine has not established APN roles, which may have helped meet care demand throughout the ongoing war with Russia. Detailed descriptions of the state of APNs in countries currently in or at risk for armed conflict are included in Supplementary Material S1.

We posit that so long as APNs are well integrated into the health system, they can minimize the health burden resulting from armed conflict (Porat‐Dahlerbruch et al. 2023a; Porat‐Dahlerbruch et al. 2025a). APN integration is defined as “a multi‐level process of incorporating APNs into a care model to the extent that they can function to their full scope of practice and education, which leads to improved patient, health system, and population needs” (Porat‐Dahlerbruch et al. 2025, p.4). Policies facilitating efficacious integration, nevertheless, must be incorporated before conflict arises because swift, effective policy development is difficult once conflict strikes (Jawad et al. 2020; Porat‐Dahlerbruch et al. 2023a). In this article, we present an evidence‐based argument for health systems to invest in APN integration and workforce expansion before armed conflict arises. We draw on an exemplar of Israel's APN policy development before and during the Israel–Hamas War.

## 2 Sources of Evidence

Based on guidance from Sukhera (2022), we performed a critical narrative review of the recent literature on three topics: (1) benefit of APNs during times of conflict, (2) systemic policy setbacks to integrating APNs into health systems prior to conflict, and (3) benefit of APNs during other public health crises. Narrative reviews can be used to describe the most relevant and significant literature supporting or refuting the authors’ perspectives in viewpoint articles (Horsley 2019). We selected a critical approach in which investigators first describe their point of view, knowledge, and experience and then conduct a noncomprehensive search to capture dominant themes from the literature (Sukhera 2022). The search strategy (Supplementary File S2) yielded sufficient thematic sufficiency—i.e., literature no longer adding significant information to the topic (Saunders et al. 2018).

We conducted analysis and interpretation according to the guide by Saunders et al. (2018). After conducting the search, we (1) discussed and evaluated the articles, (2) identified articles from recognized experts, (3) contextualized the literature within the search goals, (4) considered and discussed research supporting and refuting our points of view, (5) justified points in the literature with logic and valid evidence, (6) distinguished between fact and opinion, and (7) conducted another search to check if any new papers have been published since beginning the project (Saunders et al. 2018). All steps were conducted together by two members of the authorship team—this is acceptable for nonsystematic reviews (Sukhera 2022). Finally, we synthesized the literature by topic and wrote a discussion of our perspective, informed by the narrative review.

## 3 Results and Discussion

### 3.1 APNs and Benefits to Public Health During Armed Conflict

One reason why APNs stand out among other professionals is their focus on preventative care, facilitated by education emphasizing a patient's specific community, family, and individual factors (De Raeve et al. 2024; International Council of Nurses 2020). Patient *education*, moreover, is an effective means to increase patient *engagement* in their health (Johnson et al. 2023). Patient engagement helps achieve the quadruple aim of healthcare, which is to: (1) improve patient outcomes, (2) improve patient experience, 3) reduce costs, and (4) improve provider work experience (Johnson et al. 2023). These elements are beneficial, particularly in primary care system responses to public crises; the APN approach to care can help prevent and address chronic conditions resulting from armed conflict, such as cardiac disease, women's health, and mental health conditions (International Council of Nurses 2020; Lewis et al. 2012).

Another distinguishing feature of APN practice is a focus on holistic and humanistic care (International Council of Nurses 2020; Rosa et al. 2020). This approach is useful for responses to armed conflict when the immediate clinical needs often overlap with mental, social, and environmental needs (Lewis et al. 2012; Rosa et al. 2020). This person‐centered approach to treatment augments care, particularly when caring for vulnerable populations facing armed conflict and public health crises (Reed and Fitzpatrick 2024; Rosa et al. 2020).

### 3.2 Systemic Setbacks Preventing APNs From Contributing to Public Health Efforts in Armed Conflict

APNs are often hindered from contributing to population health needs at their maximal potential during times of war. It is critical that APN authorities and/or regulatory frameworks are established prior to the arrival of a crisis. As seen during the COVID‐19 pandemic, other viral epidemics, and recent armed conflict, it takes longer to adjust regulations and respond once a crisis arrives. In this subsection, we discuss issues affecting the integration of APNs into health systems that could be addressed to improve health system responses to future crises.

Government/jurisdictional issues typically stem from legal/regulatory scope of practice restrictions resulting from a lack of knowledge on APN care quality and educational preparation (National Academies of Sciences, Engineering, and Medicine 2021). Even the United States, the country with the longest history of a recognized APN role, still faces policy setbacks, hindering the potential for APNs to improve outcomes, mitigate public health crises, and alleviate provider shortages (Porat‐Dahlerbruch et al. 2025b; Schorn et al. 2022). In Israel, specialty‐specific shortages and resulting gaps in health service delivery have created the impetus to integrate more APNs (Fighel and Hefetz 2023). Still, systemic barriers exist for the Israeli health system as well, such as resistance from professional physician organizations (Porat‐Dahlerbruch et al. 2023b). In Israel's centralized health system, issues securing sufficient allotted positions (“tekenim”) for APNs and prerequisite APN education program requirements continue to hinder integration of the role (Porat‐Dahlerbruch et al. 2023b). Other international barriers to APN integration outside Israel and the United States include a lack of education standardization, poor understanding of APN roles, and political pushback from the medical profession (Maier et al. 2017; Porat‐Dahlerbruch et al. 2025b; Torrens et al. 2020). Internationally, there is also a lack of title uniformity, creating confusion about the APN responsibilities (Maier et al. 2017; Porat‐Dahlerbruch et al. 2022). Many of these common international barriers to APN integration stem from non‐evidence‐based notions that APNs provide lower care quality (National Academies of Sciences, Engineering, and Medicine 2021).

To combat national/jurisdictional‐level setbacks, several policy interventions are shown to be effective, including efficacious planning for APN integration, development of a standardized, national accreditation system for education, clear communication of certification standards, and appropriate financing schemes to incentivize APN employment (Maier et al. 2017; Porat‐Dahlerbruch et al. 2024; Porat‐Dahlerbruch et al. 2025b). These recommendations, however, may be less feasible for countries in the early stages of APN integration (Porat‐Dahlerbruch et al. 2023a). Nations lacking centralized workforce planning, primary care infrastructure, nursing accreditation bodies, and educational institutions are less likely to integrate APNs efficiently (Porat‐Dahlerbruch et al. 2023a). Hence, it is critical for countries leading the integration of APNs, such as the United States, Canada, and Israel, to publish research guiding best APN integration policies and practices.

### 3.3 Learning From Other Public Health Crises

The H1N1 and Ebola pandemics elucidated gaps in healthcare workforce preparedness for public health crises. However, the lessons learned were not applied to the COVID‐19 pandemic (Reed and Fitzpatrick 2024). To facilitate better preparedness, health systems can integrate more APNs so that their unique and versatile role can be optimized fully. Still, this preparation should begin now and not in an ad hoc manner after a disaster occurs (Porat‐Dahlerbruch et al. 2022).

Where APNs were well integrated into the health system during the COVID‐19 pandemic, care access and population outcomes improved (Reed and Fitzpatrick 2024). For instance, APNs created pop‐up intensive care units, vaccination, and testing sites (Reed and Fitzpatrick 2024). APNs worked among several populations, including geriatric, palliative, and pediatric populations (Rosa et al. 2020). The versatility of the APN role allowed many to be placed in new settings and were still able to provide life‐saving care and improve population health (Reed and Fitzpatrick 2024). These results can be replicated internationally during future crises, such as a viral pandemic or armed conflict, if health systems start developing policies facilitating APN integration.

### 3.4 Incorporating the International Council of Nurses Core Competencies in Disaster Nursing

The International Council of Nurses (2019) published competencies of disaster nursing, and the competencies were divided by levels: (I) general, (II) advanced/specialized nurse, and (III) disaster team leader. Countries wishing to utilize APNs in their disaster preparedness plans could model programs on Level II. In planning, the International Council of Nurses (2019) suggests working with a country's nursing education programs, national professional nursing associations, or disaster/emergency response organizations to implement education programs to prepare the APN workforce specifically for disaster/emergency response.

### 3.5 The APN Response in Israel Since the Beginning of the Israel–Hamas War: An Exemplar

Since October 7, 2023, Israel has been in a war on multiple fronts. The conflicts have required the health system to care and rehabilitate thousands with complex injuries, including hostages returning from captivity, soldiers, and thousands of citizens evacuated from their homes in war zones. The health system responded quickly by providing care for the population and was assisted by the nurse practitioner (NP) workforce. NPs are one of the most common roles and titles for APNs around the world. In Israel, NPs are authorized to prescribe and provide care in a specific specialty, such as geriatrics or neonatal care. In response to the war, NPs specializing in pain control, wound/stoma care, postoperative care, and rehabilitation were deployed. In communities, family, geriatric, and diabetes NPs were deployed.

#### 3.5.1 The Role of Existing Policy on NPs Responding to War

Several regulations enacted prior to the war facilitated a rapid response by the NP workforce. NPs in Israel did not require any changes to role authorities and descriptions to care for the wounded in hospitals and temporary clinics established for displaced populations. As such, without government intervention, NPs provided care across the country in community clinics, at home, virtually, in acute care, emergency departments, and in long‐term care. The NPs attended to the hostages returning home by addressing physical and mental health needs. Acute care included first aid, traumatic injury and amputation care, pain treatment, and mental health support. The NPs aided in rehabilitation settings by building holistic rehabilitation plans for the wounded. NPs cared for the physical needs of patients with diabetes, heart failure, lung failure, and neurological conditions. All the while, NPs were engaged in public health promotion for patients with sequelae of conflict‐related stress and anxiety. The takeaway lesson from NP deployment in Israel reflects the relatively advanced level of integration *before* the war began (Porat‐Dahlerbruch et al. 2023b). As such, ad hoc planning was not necessary, and prior policy governed APN practice without government intervention.

#### 3.5.2 Changes to NP Policy in Response to the War

The Ministry of Health leveraged its centralized planning to enact policy, creating an efficient NP workforce response. To equip NPs with skillsets to work in these fields in the short run, NPs and other health professionals led specialized training to ensure efficient workforce readiness and deployment of more providers in urgent care units, rehabilitation, and outpatient mental health. As part of a longer‐term health system response to address mental and physical population health needs from the war, the Ministry allocated funds to develop intensive NP education courses for licensing and deploying NPs specializing in psychiatry, community health, and rehabilitation.

#### 3.5.3 Centralized and Decentralized Health Systems Responding to War

Centralized health systems, generally, can fit health professions to the evolving clinical needs of the population. Hence, throughout the Israel–Hamas War, the Ministry of Health's Nursing Administration conducted constant evaluation of workforce policy, proposed amendments, and implemented changes relatively quickly, notwithstanding internal government political processes. As a specific example, the Nursing Administration adjusted the authorities and role description of postoperative and diabetes NPs in response to the war.

Indeed, Israel's centralized health system supported the efficiency of policy development and implementation. Decentralized health systems, on the other hand, are often more effective at responding to local‐level equity and resilience through decision‐making that is more fitting to a local population (Brennan and Abimola 2023). When applying APN policy development to decentralized health systems, greater decision‐making authority may facilitate APN autonomy in underserved areas—a phenomenon apparent with state‐level APN scope of practice regulations across the United States before, during, and after the COVID‐19 pandemic (Porat‐Dahlerbruch et al. 2023b; Rosa et al. 2020).

3.5.4

Israel demonstrates the benefit of developing policies now to facilitate a rapid response to population health needs when a crisis arises. It is important to note that Israel experienced relative ease adjusting policies before and during the Israel–Hamas War because of their centralized health system structure. As a result of efficacious national‐level policy, NPs addressed care for patients requiring immediate attention, namely, rehabilitation, mental health, pain management, and injury care.

## 4 Conclusion

As the frequency and intensity of conflict increase globally, the impact of APNs on public health will become increasingly critical (Reed and Fitzpatrick 2024). APNs can contribute positively to care, though policies must be in place before conflict and other crises so that the APN workforce can be deployed effectively and efficiently.

## 5 Implications for Health Policy

The future and transformation of healthcare will benefit from policymakers incorporating policies and guidelines enabling APNs to practice to their fullest capabilities during crises. However, there is a lack of evidence‐based knowledge on the benefits of integrating more APNs to improve responses to conflict (Lowe et al. 2023). It would be beneficial for governments to invest in research examining the unique role of APNs and the impact of their care during public health crises.

## Author Contributions

Conceptualization: JPD. Software: JPD. Writing—original draft: JPD, JB, and HF. Writing—review and editing: JPD, JB, and HF. Visualization: JPD. Resources: JPD. Project administration: JPD. Supervision: JPD.

## Conflict of Interest

No conflict of interest has been declared by the authors.

## Supporting information




**Supplementary Material 1**: Brief Overview of Advanced Practice Nurse Roles in Countries and Regions Currently in Armed Conflict or at Risk for Armed Conflict.

**Supplementary Material 2**: Critical Narrative Review Search Strategy and Terms.
