# Supplementary material for: Investing in the Advanced Practice Nursing Workforce to Improve Health System Responses to Armed Conflict
Source: Int Nurs Rev. 2025 Jul 18;72(3):e70074. doi: 10.1111/inr.70074 (PMC12274788; doi:10.1111/inr.70074)
Supplement: Supplementary file 1 — Supplementary Material 1: Brief Overview of Advanced Practice Nurse Roles in Countries and Regions Currently in Armed Conflict or at Risk for Armed Conflict. [file INR-72-0-s002.docx]

**Supplementary Material 1:** Brief Overview of Advanced Practice Nurse Roles in Countries and Regions Currently in Armed Conflict or at Risk for Armed Conflict

| **Country** | **Advanced Practice Nurse Roles** | **Relevant Information about the State of Advanced Practice Nurses** |
| --- | --- | --- |
| **Estonia** | Advanced practice nurse | - Advanced practice nurses have limited prescribing abilities (Sulosaari et al. 2023). - A master’s degree in nursing with a development project is required (Sulosaari et al. 2023). - Two common specializations are intensive care and mental health (Sulosaari et al. 2023). |
| **Finland** | Clinical nurse specialist  and  nurse practitioner | - Both advanced practice nurse roles are established but not formally recognized by the government, except for a minority of nurse practitioners have completed additional post-graduate education to gain medication prescriptive authorities (with physician collaboration) (De Raeve et al. 2024). - Clinical nurse specialists are educated at the master’s level to develop evidence-based nursing practice through patient care, leadership, and scholarship (Finnish Nurses Association, 2023; Jokinemi et al. 2022). - Nurse practitioners are educated at the master’s level to perform physical assessments, diagnose, and manage patients in primary care settings and acute care populations (Finnish Nurses Association, 2023; Jokinemi et al. 2022). - A lack of nurse practitioner role clarity, stemming from an absent national regulatory framework, acts as the largest barrier to the integration of nurse practitioners into the Finnish health system (Jokinemi, 2022). |
| **Gaza and the West Bank** | None | - The only documented advanced practice nursing role is ophthalmic nurse practitioners who can perform minor procedures, refer patients to physicians, and order laboratory tests (Almukhaini 2022). |
| **Iran** | Neonatal nurse practitioner | - Graduate-level education for neonatal nurse practitioners exists, though there is no prescriptive authority nor legal/regulatory framework formalizing the role (Rivaz et al. 2021). - Neonatal nurse practitioner master’s level education was established in 2009 in cooperation with Iran’s professional anesthesiologist organization (Rivaz et al. 2021). |
| **Israel** | Expert clinical nurse (often referred to as nurse practitioners in the literature) | - The Parliament in Israel sanctioned the expert clinical nurse role in 2013. Specialty areas are developing continuously in fields requiring more providers with patient-centered approaches to care to meet demand for health services (Fighel & Hefetz 2023). - Expert clinical nurse roles are growing in prominence with increasing numbers of licensed advanced practice nurses and education programs opening in university settings (Fighel & Hefetz 2023). |
| **Latvia** | None | - No education program nor role exists (De Raeve 2024). |
| **Lithuania** | Nurse practitioner | - Nurse practitioners are regulated by the government (De Raeve 2024). - Nurse practitioners can diagnose and prescribe medications if ordered previously by a physician (Sulosaari et al. 2023). - Students can specialize in primary care, anesthesiology, or intensive/acute care (Sulosaari et al. 2023). - Education occurs in a master’s program and requires a master’s thesis (Sulosaari et al. 2023). |
| **Lebanon** | None | - Advanced practice nurse roles are evolving in Lebanon, especially amid health provider shortages in the last few years (Almukhaini 2022). - A draft legal framework has been established (Almukhaini 2022). - There is an established clinical Master of Science in Nursing program (Almukhaini 2022). |
| **North Korea** | None | - No recent literature identified |
| **Poland** | None | - An advanced practice nurse role has not yet been approved legally (Kazakidis & Kryczka 2021). - The Minister of Science and Education has established graduate studies for advanced practice nursing and adopted a document laying the groundwork for advance practice nursing (Kazakidis & Kryczka 2021). Still, further educational program and legal scope of practice changes are needed to establish an advanced practice nurse role (Kazakidis & Kryczka 2021). |
| **Russia** | None | - No recent, relevant literature identified |
| **South Korea** | Advanced practice nurse | - There are 13 specializations with master’s level education and established scope of practice: adult health, anesthesia, child health, critical care, emergency care, gerontology, home health, hospice, infection control, occupational health, oncology, psychiatric-mental health, and public health (Choi et al., 2023). - Advanced practice nurses are authorized to provide care without physician collaboration in rural, underserved areas (Choi et al. 2023). - Impediments to advanced practice nurse integration include poor clarity on scopes of practice, absence of a medical fee schedule, and physician resistance to expanded nurse autonomy (Choi et al. 2023). |
| **Taiwan** | Nurse Practitioner | - Nurse practitioners can assist physicians to provide certain healthcare services (Ho et al. 2021). - Authorized healthcare services include explaining the patient's condition, consulting, ordering laboratory work, prescribing medication, and composing progress notes (Ho et al. 2021). |
| **Ukraine** | None | - Non-peer-reviewed work refers to the early establishment of “nurses with extended powers,” and describes a need to create legislative and organizational changes to implement a role (Bezkovaina 2023). |
| **Yemen** | Advanced practice nurse | - Since the 1960s, nurses commonly prescribe when there is no available physician, despite lacking regulatory/legal framework (Almukhaini 2022). - Advanced practice nurses are trained with skills and knowledge in hospital management, nutrition, and home visits (Almukhaini 2022). - A ministerial decree supports advanced practice nurse education in anesthesia, physiotherapy, and health management in the High Institute of Health Sciences (Almukhaini 2022). |

**Caption:** The information contained in this table is an overview of the state of advanced practice nurse roles in a sampling of countries/regions either in or surrounded by armed conflict. The presented information is intended to provide an impression of the state of advanced practice nursing in the selected countries rather than a comprehensive summary.
