# Supplementary material for: Investing in the Advanced Practice Nursing Workforce to Improve Health System Responses to Armed Conflict
Source: Int Nurs Rev. 2025 Jul 18;72(3):e70074. doi: 10.1111/inr.70074 (PMC12274788; doi:10.1111/inr.70074)
Supplement: Supplementary file 2 — Supplementary Material 2: Critical Narrative Review Search Strategy and Terms. [file INR-72-0-s001.docx]

Supplementary Material 2: Critical Narrative Review Search Strategy and Terms

We searched PubMed, CINAHL, Web of Science, Google Scholar, and Scopus with the following search terms organized by topics. We limited searches to the last 10 years and included any peer-reviewed articles (e.g., empirical research or commentary).

1) Benefit of APNs during time of conflict

- (“advanced practice nurses” OR “advanced practice nursing”) AND (“conflict” OR “war” OR “crisis”)

2) Systemic policy setbacks to integrating APNs into health systems prior to conflict

- “policy” AND (“setback” OR “barrier”) AND (“advanced practice nurses” OR “advanced practice nursing”) AND (“integration” OR “implementation)

3) Benefit of APNs during other public health crises

- (“benefit” OR “advantage”) AND (“advanced practice nurses” OR “advanced practice nursing”) AND (“public health crisis” OR “public health disaster”).
